# Attributed Contribution of Therapist’s Emotional Variables to Psychotherapeutic Effectiveness: A Preliminary Study

**DOI:** 10.3389/fpsyg.2021.644805

**Published:** 2021-07-29

**Authors:** Antonio Romero-Moreno, Alberto Paramio, Serafín Cruces-Montes, Antonio Zayas, Rocío Guil

**Affiliations:** Department of Psychology, Institute of Research in Social Sustainable Development (INDESS), University of Cádiz, Cádiz, Spain

**Keywords:** psychotherapeutic effectiveness, therapeutic process, common factors, psychotherapeutic attributions, emotional variables

## Abstract

**Background:**

The aim of this manuscript is to analyze the degrees of responsibility for healing that psychotherapists attribute to a set of emotional variables of the therapist involved in the therapeutic process. Such variables, framed within the well-known common factors in psychotherapy, have been proven to be essential in making the therapeutic process effective, as has been shown by research in psychotherapy in recent decades.

**Materials and Methods:**

Based on an extensive literature review, the responses from a sample of 69 psychotherapists to a tool created *ad hoc* are analyzed to verify whether their attributions are in line with the results of said review.

**Results:**

The therapists have doubts about the factors responsible for psychotherapeutic effectiveness, as well as about the value of common variables, including those of an emotional nature, not valuing them above those of a specific type. They also argue against the similar effectiveness of different psychotherapeutic models.

**Conclusion:**

Discrepancies have thus been found between the conclusions reached by research on therapeutic processes and the statements made by the therapists studied, which could indicate an insufficient impact of psychotherapeutic research on clinical practice. We also propose courses of action such as establishing training programs for the acquisition and development of emotional skills for therapists that could increase the effectiveness of their interventions.

## Introduction

Emotional variables have been shown to be fundamental to the success of psychotherapy, as shown by therapeutic process research over the last few decades. These variables are grouped into different factors, all of which are common to different types of psychotherapy, which have been identified as the main sources of psychotherapeutic effectiveness, and being more important than even the specific variables, i.e., the approach and techniques specific to each therapy (Lambert, 1992; Asay and Lambert, 1999; Safran and Muran, 2000; Lambert and Ogles, 2004; Samstag et al., 2004; Germer et al., 2005; Karson and Fox, 2010; Wampold and Imel, 2015; Botella and Maestra, 2016; Wampold, 2017; among others).

Although the technique used had traditionally been considered to have the main responsibility for the healing process (Critelli and Neumann, 1984), research on psychotherapeutic effectiveness has shown that the common variables are that which explains the greatest percentage of therapeutic change. These common variables may be related to: the patient (expectations of healing and faith in the therapist, etc.); the therapist (empathy displayed and listening skills, etc.); and the therapeutic interaction (therapeutic alliance) (Nathan et al., 2000; Wampold, 2001; Lambert and Ogles, 2004; Cuijpers et al., 2012; Norcross and Wampold, 2018; Sommers-Flanagan and Sommers-Flanagan, 2018; Eubanks and Goldfried, 2019).

The therapist’s emotional variables have also been considered to be essential elements for the establishment of a therapeutic alliance conducive to change (Keijsers et al., 2000; Ackerman and Hilsenroth, 2003; Krause, 2005; Horvarth et al., 2011; Araya-Véliz and Moncada, 2016; Araya-Véliz and Porter, 2017; Norcross and Lambert, 2018, 2019; Norcross and Wampold, 2018, 2019; Rodríguez-Morejón, 2019). Elements such as empathy, emotional intelligence and appropriate emotional adjustment and activation have been associated with a better prognosis in psychotherapy (Bohart et al., 2002; Jiménez, 2005; Santibáñez et al., 2008; Kaplowitz et al., 2011; Kolden et al., 2011; Greenberg, 2017; Hill and Castonguay, 2017; Elliott et al., 2018; Watson, 2019). In addition, in therapy, the patient learns from the therapist that they are able to master and cope with subdued or avoided emotions, thus strengthening their self-esteem. In the same way, emotions work as an activating element of the patient’s involvement with the therapist in achieving a change that allows them to overcome their problem (Frank, 1982).

Of particular interest is the classification established by Beutler et al. (2004) in which therapist variables are categorized into four blocks: (a) observable traits: gender, race, and age; (b) observable states: training, therapist experience, and directivity; (c) inferred traits: personality, empathy, well-being, and values; and (d) inferred states: the therapist’s view of the therapeutic relationship.

Among the main emotional variables of the therapist investigated, and which may contribute to therapeutic success, the following stand out.

### The Empathy Showed by the Therapist

Not only has it been related to better adherence to the patient’s treatment, but it has also been considered a fundamental element for therapeutic change (Elliott et al., 2018; Watson, 2019). Thus, Lafferty et al. (1989) found that more effective therapists show a higher level of empathic understanding than less effective therapists. According to Bohart et al. (2002), empathy correlated *r* = 0.26 with psychotherapeutic change. Rondón et al. (2009) found that the absence of empathy and the therapist’s lack of understanding of the problem was conducive to the patient’s therapeutic dropout. Therefore, it is established that an empathic attitude on the therapist’s part favors continuity of treatment (Corning et al., 2007).

### Therapist Directivity/Support

It is considered to be the degree to which instructions, information, specific help is provided, and tasks are structured and delimited (Bados and García, 2001). Traditionally, directivity and therapist support have been studied together in research on the therapeutic process (Baer et al., 1980; Lafferty et al., 1989) and are commonly regarded as qualities of an effective therapist (Navarro et al., 1987). It has been included as an emotional variable because, like empathy or patient acceptance, it can be considered as a relational and facilitative attitude of the therapist that can lead to an emotional disposition (Castillo and Poch, 1991). Different studies on directivity/support (reported in Bergin and Garfield, 1994) show a predominance of positive relationships between therapist directivity and beneficial outcomes when exercised at moderate levels. In order to obtain favorable results, a good therapist must modulate his or her directivity depending on the phase of treatment, the type of problem addressed in the consultation and the personality characteristics of the patient (Keijsers et al., 1995; Bados and García, 2001; Karmo et al., 2002; Urzúa et al., 2010), and with flexibility being considered a fundamental quality of the therapist (Rodríguez-Morejón, 2019).

### Degree of Acceptance, Understanding and Encouragement Shown by the Therapist to the Patient

This encompasses a series of aspects of the psychotherapy professional involved in the success of the therapy (Castillo and Poch, 1991). Winkler et al. (1989) suggested that the psychotherapist should have an attitude favoring a therapeutic climate that facilitates change based on respect, acceptance, understanding, warmth, and helpfulness. Such a conclusion is analogous to that established in the review by Lingiardi et al. (2018) in the field of psychodynamic therapies. Ruiz (1998) refers that therapeutic skills such as those mentioned should be present in any professional engaged in clinical practice. But it is just as important to show a willingness to listen to and understand the patient as it is to make patients feel listened to and understood (Krause, 2005). In this sense, the therapist’s ability to listen to and understand him/herself is critical (Neff, 2012) since the reactions and associations to the patient’s material are crucial information for understanding the client’s dynamics (Araya-Véliz and Moncada, 2016). Ultimately, as Bohart and Tallman (2010) state, therapists must believe in their clients’ possibilities and, from this position, listen to and privilege their experiences and ideas for change, in addition to showing unconditional acceptance toward them (Farber and Doolin, 2011).

The aim is to analyze the importance that therapists attach to these affective variables, which mainly include therapist variables such as empathy, support, and the degree of acceptance and understanding offered to the patient. We should also check whether certain conditions of the therapist, such as the therapist’s theoretical orientation or the frequency with which they access specialized publications, and affect their attributions. In this sense, with respect to theoretical orientation, we might expect eclectic psychotherapists to have a more favorable stance toward common variables, including emotional ones, given the basic tenets of their theoretical orientation, and which advocates an integration of perspectives and techniques (Norcross and Goldfried, 2005). Or that cognitive-behavioral therapists attach greater importance to directivity, as they adopt a more active and directive stance in their interventions than therapists of other orientations, such as psychoanalysts (Martorell and Prieto, 2002; Urzúa et al., 2010). On the other hand, given that most publications on research on therapeutic processes coincide in assigning common (and emotional) factors the main relevance in the healing process (Nathan et al., 2000; Lambert and Ogles, 2004; Samstag et al., 2004; Norcross, 2005; Karson and Fox, 2010; Wampold, 2017), it is to be expected that the therapists who most access these publications tend to preferentially and significantly opt for such common factors.

## Objectives and Hypotheses

Based on the above, the present study aims to clarify whether the psychotherapists have adopted the main conclusions reached by research on therapeutic processes with respect to the values of different emotional variables under study. In order to achieve this objective, the following specific objectives are also set out.

• Analyze the importance that therapists attach to the selected emotional variables.
• Verify whether certain aspects of the therapist could influence their opinion on the matter, e.g., the therapist’s theoretical approach and the frequency with which they consult specialist publications.
• Examine to what extent the results of said research have served to modify the therapists’ traditional belief held that the technique used, derived from the theoretical approach used, is the main factor responsible for the therapeutic healing process.

The hypotheses proposed as a result of the objectives stated are the following:

• H1. Contrary to the main conclusions reached by research on the therapeutic process, psychotherapists will attribute the specific variables (technique and therapeutic approach used) to explain the therapeutic change, over and above the common emotional variables.
• H2. Psychotherapists are more likely to think that not all psychotherapeutic models are similarly effective, and will attach a higher degree of efficacy to the therapeutic model they subscribe.
• H3. Eclectic psychotherapists will point to common emotional variables as primarily responsible for therapeutic change and cognitive-behavioral psychotherapists will attach greater importance to directivity than psychodynamically oriented psychotherapists.
• H4. Therapists who regularly access the psychotherapy research literature will significantly select common variables, including emotional variables, as most relevant to the therapeutic process.
• H5. Therapists favoring the similar effectiveness of different psychotherapeutic models will rate common variables (especially emotional variables) significantly higher than specific variables as the main providers of therapeutic effectiveness.

## Materials and Methods

### Participants

The population under study consisted of all the psychotherapists included in the directory of psychology clinics published by the Official Association of Psychologists of Western Andalusia (Spain). A questionnaire was administered on the attribution of psychotherapeutic effectiveness. A total of 69 individuals completed the questionnaire as a result of two invitations to participate, of which 35 were men and 34 women (50.7 and 49.3%, respectively).

The mean age of the population surveyed was 41.5 years, with a standard deviation of 6.41. In terms of their education level, 52.2% had a university degree, and 47.8% had a postgraduate/doctorate degree. Most of the individuals studied reported having more than 9 years’ experience as psychotherapists (78.3%), 14.5% had between 6 and 9 years’ experience, 4.3% had between 0 and 3 years’ experience, and only 2.9% reported having between 3 and 6 years’ experience.

The most frequently reported theoretical approach used was the cognitive-behavioral approach, covering 44.9% (31 participants) of the total population surveyed, followed by the psychodynamic approach (26.1%, 18 participants), the eclectic approach (15.9%, 11 participants), the humanistic/systemic approach (10.1%, 7 participants), and those who chose not to pronounce on their theorical orientation (3%, 2 participants).

### Instrument

An *ad hoc* survey (Table 1) on the attribution of psychotherapeutic effectiveness was administered. In order to ensure the content validity of the variables that have been described as potential agents in achieving therapeutic healing, it was necessary to identify the variables that could best serve to explain therapeutic healing from among those proposed in the literature.

**TABLE 1:** Psychotherapeutic effectiveness attribution questionnaire.

1. Gender.					
2. Age.					
3. Education.					
◯ Graduate.					
◯ Postgraduate.					
◯ PhD.					
4. Years of experience as psychotherapist.					
5. Theoretical orientation.					
◯ Psychodynamic.					
◯ Cognitive.					
◯ Behavioral.					
◯ Cognitive-behavioral.					
◯ Humanistic.					
◯ Systemic.					
◯ Eclectic.					
6. How often do you access specialized publications on psychotherapy research?					
◯ Usually.					
◯ Occasionally.					
◯ Never.					
7. We would like to know to what extent you think that each of the following variables influences the improvement of a patient undergoing psychotherapy. Please, check each item according to the following: 1 is “no influence at all” and 5 is “great influence”).					

	**1**	**2**	**3**	**4**	**5**

The therapeutic approach used (the theoretical orientation on which the psychotherapy is based)					
The techniques or procedures used					
The patient’s expectations of cure					
The patient’s involvement in the therapy					
The patient’s faith and credibility in the therapist					
The empathy shown by the therapist					
The directivity and support shown by the therapist					
The therapist’s perception of the patient’s involvement					
The therapist’s ability to influence the patient					
The degree of acceptance, interest, understanding and encouragement shown by the therapist to the patient					
The experience of the therapist					
The establishment of a therapeutic alliance between the therapist and patient.					
8. Choose from the following variables the one that you believe most influences the improvement of a patient undergoing psychotherapy (only one):					
◯ The therapeutic approach used (the theoretical orientation on which the psychotherapy is based).					
◯ The techniques or procedures used.					
◯ The patient’s expectations of cure.					
◯ The patient’s involvement in the therapy.					
◯ The patient’s faith and credibility in the therapist.					
◯ The empathy shown by the therapist.					
◯ The directivity and support shown by the therapist.					
◯ The therapist’s perception of the patient’s involvement.					
◯ The therapist’s ability to influence the patient.					
◯ The degree of acceptance, interest, understanding and encouragement shown by the therapist to the patient.					
◯ The experience of the therapist.					
◯ The establishment of a therapeutic alliance between the therapist and patient.					
9. Which of the following factors do you prefer to think is most responsible for the cure of a patient undergoing psychotherapy?					
◯ The specific factors of psychotherapies (i.e., those elements that are unique to each psychotherapy and that make them different, such as the technique used, the intervention made from the basic theoretical orientation, etc.) (*if you have selected this option, go to question 12 and continue*).					
◯ The common factors of psychotherapies (i.e., those elements that appear in common in all psychotherapies, such as patient, therapist and therapeutic relationship variables) (*if you have selected this option, go to question 11 and continue*).					
◯ Both equally (*if you have selected this option, go to question 12 and continue*).					
10. Which of the following common factors of psychotherapies do you consider most important in client improvement?					
◯ The patient’s own variables.					
◯ The therapist’s own variables.					
◯ The variables specific to the therapeutic relationship.					
11. Do you think that the different psychotherapy orientations are similarly effective?					
◯ Yes (*if you have answered this option do not answer the following question*).					
◯ No (*if you have answered this option go to the next question*).					
12. What do you think is the most effective psychotherapeutic orientation?					
◯ Psychodynamic.					
◯ Cognitive.					
◯ Behavioral.					
◯ Cognitive-behavioral.					
◯ Humanistic.					
◯ Systemic.					
◯ Eclectic.					

To this end, as a prior step to the preparation of the questionnaire, an in-depth literature review of the available research on therapeutic processes and outcomes was conducted in order to include relevant questions and items for analysis. Once the variables which had been analyzed in greater depth in the literature as potentially being responsible for healing had been selected, the procedure chosen to determine the validity of the questionnaire was expert judgment as described by Osterlind (1989). A questionnaire was sent to a selected group of experts in psychotherapies and psychological treatments who were asked to collaborate in the assessment of the degree of congruence in the assignment of the different items to the objectives proposed. A total of 12 judges answered the questionnaire. Subsequently, based on their answers, the index of congruence between each item and the objective it intended to measure (Hambleton, 1980) was calculated and the items that obtained the highest scores (*I*_ik_ > 0.5) were selected for inclusion in the instrument.

The questionnaire covered a wide range of questions, all of which were coded and closed. In the first block, the therapists’ sociodemographic characteristics and the therapists’ professional characteristics (theoretical approach used, experience, and frequency with which they consult publications on psychotherapy research) were included. The second block consisted of the core items of the questionnaire. These items corresponded to each one of the psychotherapeutic variables relevant to the healing process. The psychotherapists had to rate their level of influence on the healing process from 1 to 5 (where 1 was “does not influence the patient’s improvement at all” and 5 was “strongly influences the patient’s improvement”). These variables were the following: (1) Therapeutic approach; (2) Techniques or procedures used; (3) Patient’s expectations of healing; (4) Patient involvement; (5) Patient’s faith and trust in the therapist; (6) Therapist’s empathy; (7) Therapist’s directiveness and support; (8) Therapist’s perception of patient involvement; (9) The therapist’s ability to influence the patient; (10) Degree of acceptance, understanding, and encouragement displayed by the therapist; (11) Therapist’s experience; and (12) Establishing a therapeutic alliance. In the assessment of these variables, aspects such as the therapists’ positioning with respect to common and specific factors of psychotherapy, their choice as to which psychotherapeutic approach they considered to be most effective, and their position on the similar effectiveness of different psychotherapies were taken into account.

The three variables considered by the judges to be the most congruent with the objectives proposed were: “Therapist’s experience” with the full agreement of the judges (*I*_i__k_ = 1), “Patient’s expectations of healing” and “Patient involvement” (both with an *I*_ik_ = 0.916), with the rest reaching higher levels than the minimum required for inclusion in the questionnaire (*I*_ik_ > 0.5).

Once the validity of the questionnaire had been verified, it was necessary to determine its reliability. The value of the alpha coefficient obtained for the 12 core items was α = 0.727 for a total of 63 valid cases. Valid cases correspond to participants who answered all the questions of the questionnaire. According to George and Mallery (2003) and Field (2017), this coefficient indicates a more than satisfactory level of internal consistency for that number of items. Moreover, of the 12 items, there was no item the removal of which caused the reliability coefficient (total alpha value) to increase substantially, and all of the items were sufficiently correlated (≥0.3), so it was not necessary to remove any of them.

A principal component analysis allowed the 12 variables of the instrument to be grouped into four factors. The first two variables are considered to be specific to each psychotherapy (factor III), while the rest would be among the variables common to all psychological treatments: variables favoring the therapeutic alliance (factor I), the therapist’s emotional variables (factor II), and variables facilitating patient involvement in therapy (factor IV). Factor II, which is the subject of this study, encompassed the variables “therapist’s empathy,” “therapist’s directiveness and support,” and “degree of acceptance, understanding, and encouragement displayed by the therapist.”

### Procedure

Once the questionnaire had been designed and its validity and reliability verified, it was sent for completion, along with a cover letter, to all the psychotherapists who appeared in the directory of psychology clinics of the Official Association of Psychologists of Western Andalusia (Spain), and which amounted to a total of 134 therapists. In this letter, the principal investigator identified himself, explained the purpose of the letter and the objectives of the research, and invited them to participate.

The self-report method of questionnaire administration was used mainly for its economy, but also to avoid the bias that could be introduced by the interviewers by way of influencing their responses or while recording them. Particular emphasis was placed on the anonymity of their responses, and they were informed that once the study had been presented, they would receive feedback as a result of their collaboration. The study was conducted in compliance with the Declaration of Helsinki of 1975.

### Statistical Analyses

The frequencies and basic descriptive statistics of the variables in the questionnaire were analyzed. In addition, contingency tables were prepared and χ^2^ tests were performed between the appropriate variables in order to verify the existence of relationships between the crossed variables. For numerical variables, *t*-tests and one-way ANOVAs (with *post hoc* comparisons) were performed, and contrasts of means were conducted for both independent and related samples where the goal was to identify any potentially significant differences in the assessment of the psychotherapeutic variables. Prior to the analysis of the paired samples *t*-tests, it was verified that the correlation between the variables was greater than 0.8 as suggested in the literature for small samples (De Winter, 2013).

Data analysis and processing was performed with the software IBM statistical package for the social sciences (SPSS) v25. The statistical significance threshold was set at *p* < 0.05.

## Results

The data indicate that the group whose factors were most frequently selected as bearing the main responsibility for the therapeutic healing process was that of the common factors (39.1%). This was followed by the joint action of both types of factors (36.2%) (specific and common), while the specific factors were less frequently reported as bearing such responsibility (21.7%). However, these differences in the selection of factors responsible for healing were not statistically significant [χ^2^(2, *N* = 67) = 3.70, *p* = 0.157], nor was the difference found between the percentage of therapists who chose the common factors and that of therapists who chose the specific factors (binomial, *p* = 0.090).

The percentage of participants against the equivalence of the effectiveness of psychotherapies (73.9%) is significantly higher than the percentage of those in favor of said equivalence (23.2%) (*p* < 0.000). For the set of psychotherapists who are against such equivalence, the percentage that chose their own mode of therapy as the most effective is significantly higher than the percentage that chose a different mode (*p* < 0.000).

The mean scores for the different psychotherapeutic variables suggest that the most valued variable in terms of its contribution to therapeutic healing is “Patient involvement,” followed by “Therapeutic alliance” and “Therapist’s experience.” Among the therapist’s common variables of an emotional nature, the most valued one was “Therapist’s empathy” (Table 2).

**TABLE 2:** Mean assessments of the psychotherapeutic variables.

	***M***	***SD***
Therapeutic approach	3.81	0.974
Techniques or procedures used	4.06	0.929
The patient’s expectations of healing	3.97	0.962
Patient involvement	4.55	0.738
Patient’s faith and trust in the therapist	3.70	0.912
Therapist’s empathy	4.20	0.797
Therapist’s directiveness and support	3.58	1.032
Therapist’s perception of patient involvement	3.40	1.053
The therapist’s ability to influence the patient	4.06	0.998
Degree of acceptance, interest, understanding, and encouragement displayed by the therapist	4.00	0.970
Therapist’s experience	4.25	0.870
Establishing a therapeutic alliance	4.39	0.790

*n = 63.*

When making different comparisons regarding the assessment of each of the psychotherapeutic variables of an emotional nature, no significant differences were found with respect to “Therapist’s empathy” and “Degree of acceptance, understanding, and encouragement displayed by the therapist,” so we shall focus on the results found for the variable “Therapist’s directiveness and support” (Tables 3, 4).

**TABLE 3:** Games-Howell *post hoc* test for the variable “Directiveness and support” by theoretical approach.

					**95% CI**	
**(I) Theoretical approach (recoded)**	**(J) Theoretical approach (recoded)**	***M* (I-J)**	***SE***	**Sig.**	**Lower bound**	**Upper bound**
Psychodynamic	Cognitive-behavioral	−1.188*	0.333	0.009	–2.12	–0.26
	Humanistic/Systemic	–0.759	0.427	0.316	–1.97	0.45
	Eclectic	–0.642	0.457	0.510	–1.91	0.62
Cognitive-behavioral	Psychodynamic	1.188*	0.333	0.009	0.26	2.12
	Humanistic/Systemic	0.429	0.325	0.576	–0.60	1.46
	Eclectic	0.545	0.365	0.467	–0.52	1.61
Humanistic/Systemic	Psychodynamic	0.759	0.427	0.316	–0.45	1.97
	Cognitive-behavioral	–0.429	0.325	0.576	–1.46	0.60
	Eclectic	0.117	0.452	0.994	–1.18	1.41
Eclectic	Psychodynamic	0.642	0.457	0.510	–0.62	1.91
	Cognitive-behavioral	–0.545	0.365	0.467	–1.61	0.52
	Humanistic/Systemic	–0.117	0.452	0.994	–1.41	1.18

*Differences in means significant at the 0.05 level.

*Therapeutical approach sample sizes: psychodynamic = 18; cognitive-behavioral = 31; humanistic/systemic = 7 participants; eclectic = 11.*

**TABLE 4:** Paired samples *t*-test between the variable “Therapeutic approach” and “Techniques and procedures used,” and the rest of the common variables in eclectic-oriented therapists.

	**Paired differences**							
				**95% CI**				
	***M***	***SD***	**SEM**	**Lower**	**Upper**	***t***	**df**	**Sig.**
Therapeutic approach–Patient involvement	–1.727	1.272	0.384	–2.582	–0.873	–4.503	10	0.001
Therapeutic approach–Therapist’s empathy	–1.182	1.471	0.444	–2.170	–0.194	–2.665	10	0.024
Therapeutic approach–The therapist’s ability to influence the patient	–1.182	0.982	0.296	–1.841	–0.522	–3.993	10	0.003
Therapeutic approach–Degree of acceptance, interest, understanding, and encouragement displayed by the therapist	–0.909	1.136	0.343	–1.672	–0.146	–2.654	10	0.024
Therapeutic approach–Therapist’s experience	–1.636	1.690	0.509	–2.771	–0.501	–3.212	10	0.009
Therapeutic approach–Establishing a therapeutic alliance	–1.182	1.250	0.377	–2.022	–0.342	–3.135	10	0.011
Techniques or procedures used–Patient involvement	–1.273	1.272	0.384	–2.127	–0.418	–3.318	10	0.008
Techniques or procedures used–Therapist’s experience	–1.182	1.722	0.519	–2.338	–0.025	–2.277	10	0.046

*Only the relationships in which significant differences were observed are displayed, *n* = 11.*

The results of Levene’s test indicate that the hypothesis of equality of the variances cannot be accepted [*F*(3,61) = 3.11, *p* = 0.033, with the assumption of homoscedasticity not being fulfilled in this case]. Due to said lack of equality of variances, we shall resort to other statistics alternative to ANOVA’s *F* (Table 5) that will allow us to robustly test the hypothesis of equality of means in the assessment of the variable “Directiveness and support” between groups of therapists who use different theoretical approaches. We concluded that therapists using different approaches do not assess “Directiveness and support” in a similar way.

**TABLE 5:** Paired samples *t*-test between the variables “Therapeutic approach” and “Techniques and procedures used” and the rest of the common variables in therapists who considered different psychotherapeutic models to have similar effectiveness.

	**Paired differences**							
				**95% CI**				
	***M***	***SD***	**SEM**	**Lower**	**Upper**	***t***	**df**	**Sig.**
Therapeutic approach–Patient involvement	–1.125	1.310	0.328	–1.823	–0.427	–3.435	15	0.004
Therapeutic approach–Patient’s faith and trust in the therapist	–0.500	0.894	0.224	–0.977	–0.023	–2.236	15	0.041
Therapeutic approach–Therapist’s empathy	–1.250	0.775	0.194	–1.663	–0.837	–6.455	15	0.000
Therapeutic approach–Therapist’s directiveness and support	–0.500	0.632	0.158	–0.837	–0.163	–3.162	15	0.006
Therapeutic approach–The therapist’s ability to influence the patient	–1.000	0.926	0.239	–1.513	–0.487	–4.183	14	0.001
Therapeutic approach–Degree of acceptance, interest, understanding, and encouragement displayed by the therapist	–0.938	0.772	0.193	–1.349	–0.526	–4.858	15	0.000
Therapeutic approach–Therapist’s experience	–1.063	0.998	0.249	–1.594	–0.531	–4.259	15	0.001
Therapeutic approach–Establishing a therapeutic alliance	–1.188	1.276	0.319	–1.868	–0.507	–3.721	15	0.002
Techniques or procedures used–Patient involvement	–0.938	1.436	0.359	–1.703	–0.172	–2.611	15	0.020
Techniques or procedures used–Therapist’s empathy	–1.063	0.998	0.249	–1.594	–0.531	–4.259	15	0.001
Techniques or procedures used–The therapist’s ability to influence the patient	–0.733	1.280	0.330	–1.442	–0.025	–2.219	14	0.044
Techniques or procedures used–Degree of acceptance, interest, understanding, and encouragement displayed by the therapist	–0.750	0.856	0.214	–1.206	–0.294	–3.503	15	0.003
Techniques or procedures used–Therapist’s experience	–0.875	1.310	0.328	–1.573	–0.177	–2.671	15	0.017
Techniques or procedures used–Establishing a therapeutic alliance	–1.000	1.506	0.376	–1.802	–0.198	–2.657	15	0.018

*Only the relationships that showed significant differences are displayed, *n* = 16.*

In order to pinpoint where in particular, the differences detected are, we conducted *post hoc* comparisons (Table 3). The results show that cognitive-behavioral therapists attach more importance to directiveness and the patient’s support than do therapists with a psychodynamic approach. In consonance with this, therapists who considered cognitive-behavioral therapies to be the most effective rated the “Directiveness and support” variable significantly more highly than those who favored psychodynamic therapies [*t*(38) = 3.81, *p* < 0.000, *M* = 4, *SD* = 0.81, and *M* = 2.75, *SD* = 1.21].

Regarding the frequency with which therapists consult publications on psychotherapy research, those who regularly access these publications significantly outlined the common factors over the specific factors (52.1 and 19.5%, respectively) (*p* = 0.002). No such significant differences were found in therapists who occasionally access these publications (*p* = 1). However, therapists who consult these publications regularly give significantly less value to the variable “Directiveness and support” than those who consult them occasionally, without a fixed frequency [*t*(64) = −2.69, *p* = 0.009, *M* = 3.34, *SD* = 1.10, and *M* = 3.96, *SD* = 0.77, respectively].

Next, we will focus on whether the different groups of psychotherapists, depending on their various characteristics, differ with respect to factor II, which includes the emotional variables of therapists (“Therapist’s empathy,” “Therapist’s directiveness and support,” and “Degree of acceptance, understanding, and encouragement displayed by the therapist”).

In terms of theoretical approach, it was found that eclectically oriented therapists value factor II significantly more than factor III (specific variables). This difference favoring the factor that encompasses therapists’ emotional variables was only found in therapists with this theoretical approach.

When comparing the assessments made by eclectic therapists between the specific variable “Therapeutic approach” (*M* = 2.91, *SD* = 1.13) and the ten common variables presented, significant differences were found in six of them, always in favor of the common variables. Of those six, two are emotional: “Therapist’s empathy” and “Degree of acceptance, understanding, and encouragement displayed by the therapist.” As for the contrasts made between the other specific variable, i.e., “Techniques and procedures used” (*M* = 3.36, *SD* = 1.12) and each of the common variables, significant differences were obtained in favor of two common variables that were not of an emotional nature (Table 4).

The therapists who considered different psychotherapies to have similar effectiveness showed a clear positioning in favor of the common factors over the specific factors (binomial, *p* < 0.000). Along the same lines, we found that these therapists rate factor II and factor I (therapist’s emotional variables and alliance-building variables, respectively) significantly higher than factor III (specific variables).

Only the therapists who considered different psychotherapies to be of equivalent effectiveness presented with differences in which the common factors were more important than the totality of the psychotherapists in the study. Looking at this group of therapists in detail, when comparing the ratings between the specific variable “Therapeutic approach” (*M* = 3.25, *SD* = 0.93) and each of the common variables, eight significant differences were observed, always in favor of the common variables, including the three of an emotional nature: “Therapist’s empathy,” “Therapist’s directiveness and support,” and “Degree of acceptance, understanding, and encouragement displayed by the therapist.” In the case of the specific variable “Techniques or procedures used” (*M* = 3.44 and *SD* = 1.09), six significant differences were found, also in favor of the common variables, including two of an emotional nature: “Therapist’s empathy” and “Degree of acceptance, understanding, and encouragement displayed by the therapist” (Table 5).

In summary, of 20 comparisons made, 14 significant differences were observed favoring the common variables, including five in favor of those of an emotional nature. In the therapists who are against the similar effectiveness of psychotherapies, only eight significant differences were obtained, with three of them favoring common variables and none of those being of an emotional nature.

## Discussion

Despite the proven relevance of common variables for effective psychotherapy, especially those of an emotional nature, the present study reveals that these variables are not particularly valued by the therapists studied or not clearly preferred over variables of a specific nature. Our data seem to suggest that therapists still harbor certain doubts about the factors responsible for the effectiveness of the therapies they implement, despite what research into psychotherapeutic processes has repeatedly concluded.

Also, the study’s therapists were mostly and significantly against the similar effectiveness of the different psychotherapeutic models, giving a higher degree of effectiveness to the model they subscribe.

A more detailed analysis of emotional variables’ assessment according to the therapists’ different characteristics indicates that therapists who regularly consult specialized publications on psychotherapy research prefer common factors over specific ones. However, they attach significantly less value to the variable “Directiveness and support” than the therapists who occasionally consult such publications. Since “Directiveness and support” is a common variable, it was expected that the therapists who reported consulting said publications more frequently would have rated this variable higher than those who consult them without a fixed frequency.

Cognitive-behavioral therapists attach more importance to the directiveness and support provided to the patient during the course of psychotherapy than do psychodynamic therapists. In consonance with this, therapists who chose cognitive-behavioral therapies as the most effective rate this variable significantly higher than therapists who favor psychodynamic therapies. These results are consistent with the assumptions of cognitive-behavioral therapies, in which therapists play a more directive role in their interventions, in contrast to psychoanalytic therapies, and in which therapists avoid giving any type of advice or guidance (Martorell and Prieto, 2002).

Psychodynamic-oriented therapists had a significantly lower average for factor II (which encompasses the therapist’s emotional variables) than that of cognitive-behavioral-oriented therapists. As a result, and given that therapists of each approach tend to choose their own approach as the most effective, it is not surprising that those who chose psychodynamic therapies also present with a significantly lower average in this factor than those who chose cognitive-behavioral therapies.

As far as eclectic therapists are concerned, they are willing to rate common variables, including those of an emotional nature, and as more relevant to therapeutic healing than psychotherapy’s specific variables (techniques and approach used). This is understandable, as the theoretical assumptions of their approach are more favorable to the common variables (Norcross and Goldfried, 2005), including those of an emotional nature and factor II, which brings together these emotional variables. However, given the low number of therapists of this orientation, these data should be taken with great caution. Psychodynamic and cognitive-behavioral therapists did not rate any common emotional variable over the specific variables, nor did they rate factor II over the other factors.

In addition, the therapists who considered different psychotherapeutic models to have similar effectiveness tend toward common factors rather than specific factors, and rate the different common variables (especially those of an emotional nature) as bearing significantly more responsibility than the specific variables. These results have not been found in therapists who position themselves against the similar effectiveness of psychotherapies. This confirms the notion that assuming the equivalence of the effectiveness of the different psychotherapeutic models means accepting, as a result, the primordial value for the psychotherapeutic process of the common elements, and including those of an emotional nature, that these models share (Caro, 2018). This reasoning is based on the fact that the similar effectiveness of psychotherapies would be explained precisely by these shared elements rather than by the elements that differentiate them, a topic that has been widely discussed in the body of research on psychotherapeutic processes (Blair-Gómez, 2015; Wachtel et al., 2020).

In summary, no clear positioning can be inferred from these therapists regarding either specific or common factors as the main factors responsible for therapeutic change. It seems to be that certain doubts persist among the therapists in this study about the elements responsible for the clinical effectiveness of psychotherapy. However, when certain characteristics of psychotherapists are examined, such as their theoretical approach, their positioning on the similar effectiveness of psychotherapies, and their positioning on the most relevant common factor for healing, they behave as one would expect.

Consequently, in terms of theoretical approach, cognitive-behavioral therapists attached more importance than psychodynamic therapists to the directiveness and support provided to patients, while eclectic therapists point to the common and emotional variables as the main ones responsible for psychotherapeutic effectiveness. All these results are consistent with the premises of their respective theoretical approaches.

Regarding their position on the equivalence of the effectiveness of the different psychotherapeutic models, we also found consistent results. Only the therapists who considered different psychotherapeutic models to have similar effectiveness identify the common and emotional factors as the main causes of therapeutic change. For these therapists, acknowledging the similar effectiveness of psychotherapies means recognizing that it is the elements that psychotherapies share which makes them effective, rather than the elements that distinguish them.

However, there is one finding that runs counter to expectations: as previously mentioned, the therapists who report consulting publications on research in psychotherapy on a regular basis rate the common variable “Directiveness and support” less highly than those who consult them only occasionally.

The present study’s major limitation is the size of the sample, so the present research has to be considered a preliminary exploratory study, pending replication with larger samples of psychotherapists to confirm the findings. Nevertheless, recommendations from the literature were followed to ensure the validity of the analyses for small samples (De Winter, 2013; Shingala and Rajyaguru, 2015). It would also be interesting to further investigate the questionnaire’s internal structure, both in larger samples and in populations with more varied psychotherapeutic orientations. On the other hand, it would be essential to include many therapist variables in future studies, such as emotional intelligence or emotional reactions toward patients. Finally, given the preliminary nature of the present study, several aspects that may influence the therapists’ attributions, such as the type of pathology mostly treated, as well as any other patient-related variable that would increase the stability of the proposed factor structure and the generalizability of the findings, have not been considered.

In short, although there is a need for further studies with a larger population of therapists in order to confirm our findings, there were discrepancies between the statements from the therapists in our study as a whole and the conclusions reached by the body of research on therapeutic processes, especially with regard to the role of common and emotional factors. In this line, as noted by Beitman (1987), research in psychotherapy seems to have insufficient impact on clinical practice. Even though research highlights the great relevance of these factors as one of the main reasons for therapeutic change, the therapists in our study still have doubts about them. In this sense, it would be interesting to develop training programs that enable therapists, regardless of their underlying theoretical framework, to acquire and develop emotional skills, such as empathy, listening skills, and emotional self-regulation, among others.

## Data Availability Statement

The raw data supporting the conclusions of this article will be made available by the authors, without undue reservation.

## Ethics Statement

Ethical review and approval was not required for the study on human participants in accordance with the local legislation and institutional requirements. The patients/participants provided their written informed consent to participate in this study.

## Author Contributions

AR-M: PI and theoretical approach. AP: methods, data analysis, and results. SC-M and AZ: discussion and research support. RG: senior researcher supervision. All authors contributed to the article and approved the submitted version.

## Conflict of Interest

The authors declare that the research was conducted in the absence of any commercial or financial relationships that could be construed as a potential conflict of interest.

## Publisher’s Note

All claims expressed in this article are solely those of the authors and do not necessarily represent those of their affiliated organizations, or those of the publisher, the editors and the reviewers. Any product that may be evaluated in this article, or claim that may be made by its manufacturer, is not guaranteed or endorsed by the publisher.
